# The Discontinuous Galerkin Finite Element Method for Solving the MEG and the Combined MEG/EEG Forward Problem

**DOI:** 10.3389/fnins.2018.00030

**Published:** 2018-02-02

**Authors:** Maria Carla Piastra, Andreas Nüßing, Johannes Vorwerk, Harald Bornfleth, Robert Oostenveld, Christian Engwer, Carsten H. Wolters

**Affiliations:** ^1^Institute for Biomagnetism and Biosignalanalysis, University of Münster, Münster, Germany; ^2^Institute for Computational and Applied Mathematics, University of Münster, Münster, Germany; ^3^Scientific Computing and Imaging Institute, University of Utah, Salt Lake City, UT, United States; ^4^BESA GmbH, Gräfelfing, Germany; ^5^Donders Institute, Radboud University, Nijmegen, Netherlands; ^6^NatMEG, Department of Clinical Neuroscience, Karolinska Institutet, Stockholm, Sweden; ^7^Cluster of Excellence EXC 1003, Cells in Motion, CiM, University of Münster, Münster, Germany

**Keywords:** discontinous Galerkin, finite element methods, conservation properties, magnetoencephalography (MEG), electroencephalography (EEG), dipole, subtraction method, realistic head modeling

## Abstract

In Electro- (EEG) and Magnetoencephalography (MEG), one important requirement of source reconstruction is the forward model. The continuous Galerkin finite element method (CG-FEM) has become one of the dominant approaches for solving the forward problem over the last decades. Recently, a discontinuous Galerkin FEM (DG-FEM) EEG forward approach has been proposed as an alternative to CG-FEM (Engwer et al., 2017). It was shown that DG-FEM preserves the property of *conservation of charge* and that it can, in certain situations such as the so-called *skull leakages*, be superior to the standard CG-FEM approach. In this paper, we developed, implemented, and evaluated two DG-FEM approaches for the MEG forward problem, namely a conservative and a non-conservative one. The *subtraction approach* was used as source model. The validation and evaluation work was done in statistical investigations in multi-layer homogeneous sphere models, where an analytic solution exists, and in a six-compartment realistically shaped head volume conductor model. In agreement with the theory, the conservative DG-FEM approach was found to be superior to the non-conservative DG-FEM implementation. This approach also showed convergence with increasing resolution of the hexahedral meshes. While in the EEG case, in presence of skull leakages, DG-FEM outperformed CG-FEM, in MEG, DG-FEM achieved similar numerical errors as the CG-FEM approach, i.e., skull leakages do not play a role for the MEG modality. In particular, for the finest mesh resolution of 1 mm sources with a distance of 1.59 mm from the brain-CSF surface, DG-FEM yielded mean topographical errors (relative difference measure, RDM%) of 1.5% and mean magnitude errors (MAG%) of 0.1% for the magnetic field. However, if the goal is a combined source analysis of EEG and MEG data, then it is highly desirable to employ the same forward model for both EEG and MEG data. Based on these results, we conclude that the newly presented conservative DG-FEM can at least complement and in some scenarios even outperform the established CG-FEM approaches in EEG or combined MEG/EEG source analysis scenarios, which motivates a further evaluation of DG-FEM for applications in bioelectromagnetism.

## 1. Introduction

Together with electroencephalography (EEG), magnetoencephalography (MEG) is a technique used to investigate brain activity. EEG and MEG are devoted to detect the electric potential distribution and the magnetic field generated by the brain, respectively, with a unique time resolution (Brette and Destexhe, 2012). An important topic in many applications of EEG and MEG is the source reconstruction, i.e., the identification of the sources in the brain responsible for the signals recorded at the head surface (EEG) or in a small distance from the head surface (MEG). Moreover, it has been shown (Fuchs et al., 1998; Aydin et al., 2015, 2017) that combined MEG/EEG employs the complementary information of both modalities providing source reconstructions that outperform the ones provided by each single modality. In order to compute MEG/EEG source reconstructions, i.e., to solve a related ill-posed inverse problem of MEG/EEG, the forward problem has to be solved. Since the accuracy of MEG/EEG inverse problem solutions depends strongly on the forward solution, it is fundamental to increase the accuracy of the latter (Brette and Destexhe, 2012). Furthermore, in a multi-modal MEG/EEG reconstruction it is desirable to use the same forward model for both EEG and MEG data. In the EEG case, the forward problem consists of the evaluation of the electric potential generated by a source located in the brain by solving an elliptic partial differential equation of second order (Wolters et al., 2007). In the MEG case, the magnetic field needs to be computed exploiting Biot-Savart's law, which depends on the EEG solution (Brette and Destexhe, 2012). In simplified scenarios, such as multi-layer sphere models with piecewise homogeneous conductivity, analytical solutions are available (Brette and Destexhe, 2012). In more realistic scenarios, e.g., realistically shaped head models, numerical methods have to be adopted. There is a large variety of numerical methods that can be employed, among them are boundary element methods (Mosher et al., 1999; Acar and Makeig, 2010; Gramfort et al., 2011; Stenroos and Sarvas, 2012), finite volume methods (Cook and Koles, 2006), finite difference methods (Wendel et al., 2008; Vatta et al., 2009; Montes-Restrepo et al., 2014) and finite element methods (FEMs) (Bertrand et al., 1991; Marin et al., 1998; Schimpf et al., 2002; Drechsler et al., 2009; Nüßing et al., 2016; Pursiainen et al., 2016). In this work, we deal with FEMs, which have shown high numerical accuracies with the possibility to model complex geometries and bioelectromagnetic properties (e.g., anisotropic conductivity) of the head. In more realistic simulations, an aspect that should be more carefully studied is the fulfilling of the conservation of charge law and its implications on the application at hand. For the EEG, this has been studied in Engwer et al. (2017), where it was shown that the phenomenon of *skull leakages*, which occur as a consequence of violating the conservation of charge law, can be overcome by using a discontinuous Galerkin FEM (DG-FEM) instead of a classical, continuous Galerkin FEM (CG-FEM). Leakage effects occur when a low conductive compartment of the head, i.e., the skull, is modeled too coarsely. This leads to scalp and cerebrospinal fluid elements being erroneously connected via single skull vertices or edges, a frequent case when segmenting, for example, children heads with thin skull compartments. Note that such skull leakage effects can also compromise the accuracy of transcranial electrical stimulation simulations (Miranda et al., 2006; Datta et al., 2013; Windhoff et al., 2013; Wagner et al., 2014), in a reciprocal sense (Wagner et al., 2016). As a further motivation, DG-FEM provides the basis for the implementation of a further improved method, the so-called unfitted discontinuous Galerkin FEM (Nüßing et al., 2016). This method combines the advantages of using a hexahedral mesh, whose generation pipeline is less complex than for the tetrahedral case, with a smooth representation of head tissue compartments.

In this work, we introduce the first application of DG-FEM for solving the MEG forward problem. As MEG solutions depend on EEG solutions, we implement a setup where the same method (CG- or DG-FEM) is adopted for both modalities, allowing for a combined EEG and MEG source reconstruction study. We analyze the accuracy of DG-FEM results in comparison to the CG-FEM results and investigate the propagation of the effects of conservation properties on the MEG results, as these effects have been proven to play an important role for EEG forward simulations in leaky scenarios. We will show that, in contrast to the EEG, the accuracy of the forward solution for the MEG is basically not affected by skull leakage effects. In fact, the accuracy of DG-FEM forward modeling is in the same range as for standard CG-FEM. However, because of the advantages on the EEG side, DG-FEM is an interesting new approach for combined MEG/EEG source reconstruction scenarios.

## 2. Theory

In this section, after a summary of the EEG and MEG background, the theory of Discontinuous Galerkin- (DG-) FEM for solving the MEG forward problem will be presented. As the MEG forward problem is build on the solution of the EEG forward problem, a brief section will be about the latter.

### 2.1. Background

Following Hämäläinen et al. (1993) and Brette and Destexhe (2012), the electric potential distribution and the resulting magnetic induction generated in the brain can be modeled through the quasistatic approximation of Maxwell's equations, when assuming that the permeability of the tissue in the head is that of the free space, i.e., μ = μ_0_,

(1a) $$\begin{array}{ll} \nabla \times \text{E} & =0, \end{array}$$

(1b) $$\begin{array}{ll} \nabla \cdot \text{E} & =\frac{\rho }{\epsilon_{0}}, \end{array}$$

related to the electrical part, and

(2a) $$\begin{array}{ll} \nabla \times \text{B} & =\mu_{0}\text{j}, \end{array}$$

(2b) $$\begin{array}{ll} \nabla \cdot \text{B} & =0, \end{array}$$

related to the magnetic part.

In Equation (2a) **j** represents the total current density produced by neuronal activity, which, in bioelectromagnetism (Hämäläinen et al., 1993; Brette and Destexhe, 2012), is split into two contributions,

(3) $$\begin{array}{l} \text{j}\left(\text{r}\right)={\text{j}}^{p}\left(\text{r}\right)+{\text{j}}^{s}\left(\text{r}\right), \end{array}$$

where **j**^*p*^ is the so called primary current, **j**^*s*^ the secondary or volume current and **r** ∈ ℝ^3^. In neuromagnetism, the primary current is widely represented as a *mathematical point dipole* (De Munck et al., 1988; Murakami and Okada, 2006),

(4) $$\begin{array}{l} {\text{j}}^{p}\left(\text{r}\right)=\text{M}\cdot \delta \left(\text{r}-{\text{r}}_{0}\right), \end{array}$$

where **M** ∈ ℝ^3^ stands for the dipolar moment and δ is the Dirac delta distribution, centered in the dipole position ${\text{r}}_{0}\in {\mathbb{R}}^{3}$. The volume current is a passive current that is the result of the macroscopic electric field on charge carriers in the conducting medium (Hämäläinen et al., 1993; Brette and Destexhe, 2012), and

(5) $$\begin{array}{l} {\text{j}}^{s}=\sigma \text{E} \end{array}$$

holds true (Ohm's law), where σ indicates the conductivity profile of the conductive medium. While, for the mathematical point dipole, the primary current is present only at the source position, the secondary current flows passively everywhere in the medium.

#### 2.1.1. The forward problem of EEG

To derive the EEG forward problem, we have to consider Equations (1a) and (2a). From Equation (1a) we deduce that there exists a potential *u* such that

(6) $$\begin{array}{l} \text{E}=-\nabla u, \end{array}$$

so that Equation (5) can be written as

(7) $$\begin{array}{l} {\text{j}}^{s}=-\sigma \nabla u. \end{array}$$

Applying the divergence to Equation (2a), we obtain

(8) $$\begin{array}{l} \nabla \cdot \text{j}=0. \end{array}$$

Combining Equations (3), (7), and (8), we get an inhomogeneous Poisson equation that, together with the homogeneous Neumann boundary condition, models the EEG forward problem:

(9) ∇·(σ∇u)=∇·jp,in Ω⊆ℝ3

(10) $$\begin{array}{ll} \sigma \nabla u\cdot \text{n}=0, & \text{on}\partial \Omega \end{array}$$

where Ω is the volume conductor and **n** is the unit outer normal vector on ∂Ω.

#### 2.1.2. Conservation properties

A fundamental physical property of the EEG forward problem is the conservation of charge:

(11) $$\begin{array}{l} \int_{\partial K}{\text{j}}^{s}\cdot \text{n}\;ds=\int_{K}f\;dK,\forall K\subset \Omega , \end{array}$$

where *f* = −∇ · **j**^*p*^ and *K* is a control volume in Ω.

For FEMs this property carries over to the discrete solution only if the test space contains the characteristic function, which is one in *K* and zero everywhere else. In general, a conforming discretization, like CG-FEM, does not guarantee this property, while the DG-FEM fulfills a discrete analog, see Remark 3.

#### 2.1.3. The forward problem of MEG

The solution of the MEG forward problem consists in the computation of the magnetic induction (flux), Φ, generated by a dipolar source in the brain. The magnetic flux is computed from the magnetic field **B** (B-field):

(12) $$\begin{array}{l} \Phi =\int_{S}\text{B}\cdot d\text{s}, \end{array}$$

where *S* is the surface of the sensor.

Furthermore, following Biot-Savart's law, the B-field at a point **r** ∈ ℝ^3^ outside the domain Ω can be computed as

(13) $$\begin{array}{l} \text{B}\left(\text{r}\right)=\frac{\mu_{0}}{4\pi }\int_{\Omega }\text{j}\left({\text{r}}^{\prime }\right)\times \frac{\text{r}-{\text{r}}^{\prime }}{|\text{r}-{\text{r}}^{\prime }{|}^{3}}d^{3}{\text{r}}^{\prime }, \end{array}$$

(Hämäläinen et al., 1993; Brette and Destexhe, 2012).

When combining Equations (3), (13), and (4), one obtains (Hämäläinen et al., 1993; Brette and Destexhe, 2012):

(14) $$\begin{array}{l} \text{B}(\text{r})\overset{\left(3,13\right)}{=}\frac{\mu_{0}}{4\pi }\int_{\Omega }\left({\text{j}}^{p}(r^{\prime })+{\text{j}}^{s}(r^{\prime })\right)\times \frac{\text{r}-r^{\prime }}{|\text{r}-r^{\prime }{|}^{3}}d^{3}r^{\prime }\\ \;\overset{\left(4\right)}{=}\frac{\mu_{0}}{4\pi }\text{M}\times \frac{\text{r}-{\text{r}}_{0}}{|\text{r}-{\text{r}}_{0}{|}^{3}}-\frac{\mu_{0}}{4\pi }\int_{\Omega }\sigma \nabla u(r^{\prime })\times \frac{\text{r}-r^{\prime }}{|\text{r}-r^{\prime }{|}^{3}}d^{3}r^{\prime }\\ \;={\text{B}}^{p}(\text{r})+{\text{B}}^{s}(\text{r}). \end{array}$$

Namely, the B-field can be split into two contributions as well, the primary B-field **B**^*p*^, which is calculated analytically for a mathematical point dipole in Equation (13), and the secondary B-field **B**^*s*^, which has to be computed numerically when the electrical potential is computed numerically (since it depends on the electrical potential *u* inside the domain Ω).

#### 2.1.4. The forward problem of MEG for multi-layer homogeneous sphere model

In simplified geometries, similarly to the EEG forward problem (Brette and Destexhe, 2012), there exist analytical solutions for the MEG forward problem (Sarvas, 1987; Ilmoniemi, 1995). Sarvas (1987) showed that the magnetic field outside a spherically symmetric conductor due to internal current sources does not depend on the profile of conductivity along the radius. He derived the following analytical MEG solution for a multi-layer homogeneous sphere model:

(15) $$\begin{array}{l} \text{B}\left(\text{r}\right)=\frac{\mu_{0}}{4\pi F^{2}}\left(F\text{M}\times {\text{r}}_{0}-\text{M}\times {\text{r}}_{0}\cdot \text{r}\nabla F\right), \end{array}$$

where **a** = **r** − **r**_0_, *a* = |**a**|, *r* = |**r**|, $F=a\left(ra+r^{2}-\text{r}\cdot {\text{r}}_{0}\right)$ and $\nabla F=\left(r^{-1}a^{2}+a^{-1}\text{a}\cdot \text{r}+2a+2r\right)\text{r}-\left(a+2r+a^{-1}\text{a}\cdot \text{r}\right){\text{r}}_{0}$.

Ilmoniemi (1995) could even demonstrate that radial anisotropy added to a spherically symmetric conductor does not affect the external magnetic field due to internal sources.

From Equation (15), one can deduce three important features of the analytical MEG solution for a multi-layer homogeneous sphere model and a point outside the model:

**Remark 1**. *Three main properties of analytical MEG solution for a multi-layer homogeneous sphere model and a measurement point outside the model (Sarvas, 1987):*

1. the solution does not depend on the conductivity profile of the spherical model
2. if the source is radial, then the B-field outside Ω vanishes
3. *the normal projection of the secondary component of the B-field gives a null contribution to the total B-field, i.e., **B**^*s*^(**r**) · **n** =* 0, *for **r** outside Ω (Sarvas, 1987).*

### 2.2. The EEG forward problem

In this section we will recall the concepts of CG- and DG-FEM for the EEG forward problem that are then needed in section 2.3 for the derivation of the two FEM based MEG forward approaches.

#### 2.2.1. The subtraction approach

The mathematical point dipole model introduces a singularity on the right hand side of the PDE in Equation (9) that can be treated with the so-called subtraction approach (Bertrand et al., 1991; Awada et al., 1997; Marin et al., 1998; Wolters et al., 2007; Drechsler et al., 2009). The subtraction approach assumes that a non-empty neighborhood Ω^∞^ around the source in **r**_0_ can be found with homogeneous conductivity σ^∞^. The conductivity tensor σ is then split into two parts,

(16) $$\begin{array}{l} \sigma =\sigma^{\infty }+\sigma^{corr}, \end{array}$$

where σ^*corr*^ vanishes in Ω^∞^. The potential *u* can also be split into two contributions,

(17) $$\begin{array}{l} u=u^{\infty }+u^{corr}. \end{array}$$

The so-called *singularity potential* *u*^∞^ is the solution of the Poisson equation in an unbounded and homogeneous conductor with constant conductivity σ^∞^, and it can be computed analytically. The *correction potential* *u*^*corr*^ becomes the unknown of a new Poisson equation:

(18) -∇·(σ∇ucorr)=∇·(σcorr∇u∞),in Ω⊆ℝ3

(19) $$\begin{array}{ll} \sigma \nabla u^{corr}\cdot \text{n}=-\sigma \nabla u^{\infty }\cdot \text{n}, & \text{on}\partial \Omega \end{array}$$

after embedding Equations (16) and (17) in Equations (9) and (10).

The conforming weak formulation of (18) and (19) presented in Wolters et al. (2007) reads: Find $u_{h}^{corr}\in W_{h}\subset H^{1}$ such that

(20) $$\begin{array}{l} \int_{\Omega }\sigma \nabla u_{h}^{corr}\cdot \nabla v_{h}dx=-\int_{\Omega }\sigma^{corr}\nabla u^{\infty }\cdot \nabla v_{h}dx\\ \;-\int_{\partial \Omega }\sigma^{\infty }\nabla u^{\infty }\cdot \text{n}v_{h}ds \end{array}$$

holds true, ∀*v*_*h*_ ∈ *W*_*h*_. Choosing *W*_*h*_ as the space of piecewise linear, continuous functions give the classical CG-FEM.

The subtraction approach is theoretically well understood. A deep numerical analysis of the subtraction approach including proofs for uniqueness and existence has been carried out in Wolters et al. (2007) and Drechsler et al. (2009).

#### 2.2.2. DG-FEM for the EEG forward problem

A discontinuous Galerkin- (DG-) FEM forward modeling approach has recently been proposed for the EEG by Engwer et al. (2017). In order to prepare our DG-FEM derivation for the MEG forward problem in section 2.3, we now recall some main properties of DG-FEM for the EEG. First, we introduce a volume triangulation $T_{h}\left(\Omega \right)$, which is a a finite collection of disjoint and open subsets forming a partition of Ω, where *h* corresponds to the mesh-width. Furthermore, the triangulation induces the *internal skeleton*

(21) $$\begin{array}{l} \Gamma_{int}:=\left\{\gamma_{e,f}=\partial E_{e}\cap \partial E_{f}|E_{e},E_{f}\in T_{h}\left(\Omega \right),E_{e}\neq E_{f},|\gamma_{e,f}|>0\right\} \end{array}$$

and the *skeleton* Γ: = Γ_*int*_ ∪ ∂Ω. Let $V_{h}^{l}$ be the so-called *broken polynomial space*, that is defined as piecewise polynomial space on the partition $T_{h}\left(\Omega \right)$:

(22) $$\begin{array}{l} V_{h}^{l}:=\left\{v\in L^{2}\left(\Omega \right):v{|}_{E}\in P^{l}\left(E\right),\forall E\in T_{h}\left(\Omega \right)\right\}, \end{array}$$

where *P*^*l*^ denotes the space of polynomial functions of degree *l* ∈ ℕ. They describe functions that exhibit element-wise polynomial behavior but may be discontinuous across element interfaces. In the following we will assume that σ is constant on each element *E*_*i*_ and denote its value by σ_*i*_.

Furthermore we recall the definition of *jump* of a function *u* on the intersection between two elements *E*_*e*_ and *E*_*f*_ of the triangulation $T_{h}\left(\Omega \right)$ with outer normal ${\text{n}}_{e}\in {\mathbb{R}}^{3}$ and ${\text{n}}_{f}\in {\mathbb{R}}^{3}$, respectively:

(23) $$\begin{array}{l} ⟦u⟧:=u{|}_{E_{e}}{\text{n}}_{e}+u{|}_{E_{f}}{\text{n}}_{f}\in {\mathbb{R}}^{3}. \end{array}$$

Note that the normals **n**_*e*_ and **n**_*f*_ are opposing vectors, i.e., **n**_*e*_ = −**n**_*f*_. In addition, the *weighted average* of *u* on the interface is defined as

(24) $$\begin{array}{l} \left\{u\right\}:=\frac{\sigma_{f}}{\sigma_{e}+\sigma_{f}}u{|}_{E_{e}}+\frac{\sigma_{e}}{\sigma_{e}+\sigma_{f}}u{|}_{E_{f}}. \end{array}$$

The DG-FEM for solving Equations (18) and (19) then reads: Find $u_{h}^{corr}\in V_{h}^{l}$ such that

(25) $$\begin{array}{l} a\left(u_{h}^{corr},v_{h}\right)+J\left(u_{h}^{corr},v_{h}\right)=l\left(v_{h}\right),\forall v_{h}\in V_{h}^{l}, \end{array}$$

with

$$\begin{array}{l} a\left(u_{h}^{corr},v_{h}\right)=\int_{\Omega }\sigma \nabla u_{h}^{corr}\cdot \nabla v_{h}dx-\int_{\Gamma_{int}}\left\{\sigma \nabla u_{h}^{corr}\right\}\cdot ⟦v_{h}⟧ds\\ -\int_{\Gamma_{int}}\left\{\sigma \nabla v_{h}\right\}\cdot ⟦u_{h}^{corr}⟧ds,\\ J\left(u_{h}^{corr},v_{h}\right)=\eta \int_{\Gamma_{int}}\frac{{\hat{\sigma }}_{\gamma }}{h_{\gamma }}⟦u_{h}^{corr}⟧\cdot ⟦v_{h}⟧ds, \end{array}$$

and

(26) $$\begin{array}{l} l\left(v_{h}\right)=-\int_{\Omega }\sigma^{corr}\nabla u^{\infty }\cdot \nabla v_{h}dx-\int_{\partial \Omega }\sigma^{\infty }\nabla u^{\infty }\cdot \text{n}v_{h}ds\\ +\int_{\Gamma_{int}}\left\{\sigma^{corr}\nabla u^{\infty }\right\}\cdot ⟦v_{h}⟧ds, \end{array}$$

where *h*_γ_ and ${\hat{\sigma }}_{\gamma }$ denote local definitions of the mesh width and the electric conductivity on an edge γ, respectively; while η is a penalty parameter.

If Equation (25) has been solved toward the correction potential $u_{h}^{corr}$, then the full potential *u*_*h*_ can be computed as $u_{h}=u_{h}^{corr}+u^{\infty }$.

**Remark 2**. *(Discrete Properties) The proposed discretization Equation (25) is consistent and adjoint-consistent with the strong problem Equations (18) and (19), and for a sufficiently large constant η* > 0 *it has a unique solution.*

**Remark 3**. *(Conservation Property) For any control volume $K\in T_{h}\left(\Omega \right)$, Equation (25) fulfills a discrete conservation property*

(27) $$\begin{array}{l} \int_{\partial K}{\text{j}}_{h}^{corr,DG}\cdot \text{n}ds=\int_{K}f^{corr}dx, \end{array}$$

*with the discrete electric flux ${\text{j}}_{h}^{corr,DG}=\left\{\sigma \nabla u_{h}^{corr}\right\}-\eta \frac{{\hat{\sigma }}_{\gamma }}{h_{\gamma }}⟦u_{h}^{corr}⟧$ and *f*^*corr*^ = −∇ · σ^*corr*^∇*u*^∞^. For *h* →* 0, *the jump $⟦u_{h}^{corr}⟧$ vanishes and the discrete flux ${\text{j}}_{h}^{corr,DG}$ converges to the flux* ***j***^*corr*^ = σ∇*u*^*corr*^.

As we will see in more detail in section 2.3.3, for the MEG problem the main quantity of interest is the electric flux, needed to compute the B-field. This flux, again, is closely related to the conservation of charge property.

### 2.3. The MEG forward problem

In this section, CG- and DG-FEM formulations for the MEG forward problem are derived, when a conservative or a non-conservative flux expression is adopted along with the subtraction approach.

#### 2.3.1. The subtraction approach in the MEG case

In this section, we will focus on the expression of the secondary B-field **B**^*s*^, as the primary B-field **B**^*p*^ is analytically computable.

When inserting Equation (17) into Biot-Savart's law Equation (14), we obtain the following expression for the secondary B-field:

(28) $$\begin{array}{l} {\text{B}}^{s}\left(\text{r}\right)=-\frac{\mu_{0}}{4\pi }\int_{\Omega }\left(\sigma \nabla \left(u^{\infty }+u^{corr}\right)\left({\text{r}}^{\prime }\right)\right)\times \frac{\text{r}-{\text{r}}^{\prime }}{|\text{r}-{\text{r}}^{\prime }{|}^{3}}d^{3}{\text{r}}^{\prime }\\ \;=-\frac{\mu_{0}}{4\pi }\int_{\Omega }\sigma \nabla u^{\infty }\left({\text{r}}^{\prime }\right)\times \frac{\text{r}-{\text{r}}^{\prime }}{|\text{r}-{\text{r}}^{\prime }{|}^{3}}d^{3}{\text{r}}^{\prime }\\ \;-\frac{\mu_{0}}{4\pi }\int_{\Omega }\sigma \nabla u^{corr}\left({\text{r}}^{\prime }\right)\times \frac{\text{r}-{\text{r}}^{\prime }}{|\text{r}-{\text{r}}^{\prime }{|}^{3}}d^{3}{\text{r}}^{\prime }\\ \;={\text{B}}_{\infty }^{s}\left(\text{r}\right)+{\text{B}}_{corr}^{s}\left(\text{r}\right). \end{array}$$

Both ${\text{B}}_{\infty }^{s}$ and ${\text{B}}_{corr}^{s}$ are computed by numerical integration on the volume Ω.

Since we are dealing with numerical integration, both *u*^∞^ and *u*^*corr*^ are projected in a discrete space, *W*_*h*_, i.e.,

(29) $$\begin{array}{l} u_{h}^{\infty }=\underset{i}{\sum }u_{i}^{\infty }\varphi_{i}, \end{array}$$

and

(30) $$\begin{array}{l} u_{h}^{corr}=\underset{i}{\sum }u_{i}^{corr}\varphi_{i}, \end{array}$$

where (φ_*i*_)_*i*_ represent a basis of the discrete space *W*_*h*_, while $u_{h}^{\infty }$ and $u_{h}^{corr}$ are the discrete representations of *u*^∞^ and *u*^*corr*^, respectively. Note that *u*^∞^ has an analytical expression. The description of the discretization process in both the CG- and DG-FEM schemes is the content of the following sections.

#### 2.3.2. CG-FEM MEG forward problem

In a CG-FEM approach the following expression of the electric flux (${\text{j}}_{h}^{corr,CG}$) is considered:

(31) $$\begin{array}{l} {\text{j}}_{h}^{corr,CG}=\sigma \nabla u_{h}^{corr}\\ \;=\underset{i}{\sum }u_{i}^{corr}\nabla \varphi_{i}. \end{array}$$

where (φ_*i*_)_*i*_ is a collection of *hat functions*, basis of *W*_*h*_.

The discretization of ${\text{B}}_{\infty }^{s}$ and ${\text{B}}_{corr}^{s}$ (i.e., ${\text{B}}_{\infty ,h}^{s}$ and ${\text{B}}_{corr,h}^{s}$) are then:

(32) $$\begin{array}{l} {\text{B}}_{\infty ,h}^{s}\left(\text{r}\right)=-\frac{\mu_{0}}{4\pi }\underset{i}{\sum }u_{i}^{\infty }\int_{\Omega }\sigma \nabla \varphi_{i}\left({\text{r}}^{\prime }\right)\times \frac{\text{r}-{\text{r}}^{\prime }}{|\text{r}-{\text{r}}^{\prime }{|}^{3}}d^{3}{\text{r}}^{\prime }, \end{array}$$

and

(33) $$\begin{array}{l} {\text{B}}_{corr,h}^{s}\left(\text{r}\right)=-\frac{\mu_{0}}{4\pi }\underset{i}{\sum }u_{i}^{corr}\int_{\Omega }\sigma \nabla \varphi_{i}\left({\text{r}}^{\prime }\right)\times \frac{\text{r}-{\text{r}}^{\prime }}{|\text{r}-{\text{r}}^{\prime }{|}^{3}}d^{3}{\text{r}}^{\prime }, \end{array}$$

respectively. Note that ${\left(u_{i}^{corr}\right)}_{i}$ are given from the EEG forward computation.

If we call **c**_*n*_ the center of the *n*th coil, then the discrete ${\text{B}}_{\infty ,h}^{s}$ and ${\text{B}}_{corr,h}^{s}$ evaluated in **c**_*n*_ are:

(34) $$\begin{array}{l} {\text{B}}_{\infty ,h}^{s}\left({\text{c}}_{n}\right)=\underset{i}{\sum }u_{i}^{\infty }\underset{: =S_{ni}^{\infty }}{\underset{︸}{\left(-\frac{\mu_{0}}{4\pi }\right)\int_{\Omega }\sigma \nabla \varphi_{i}\left({\text{r}}^{\prime }\right)\times \frac{{\text{c}}_{n}-{\text{r}}^{\prime }}{|{\text{c}}_{n}-{\text{r}}^{\prime }{|}^{3}}d^{3}{\text{r}}^{\prime }}}, \end{array}$$

and

(35) $$\begin{array}{l} {\text{B}}_{corr,h}^{s}\left({\text{c}}_{n}\right)=\underset{i}{\sum }u_{i}^{corr}\underset{: =S_{ni}^{corr,CG}}{\underset{︸}{\left(-\frac{\mu_{0}}{4\pi }\right)\int_{\Omega }\sigma \nabla \varphi_{i}\left({\text{r}}^{\prime }\right)\times \frac{{\text{c}}_{n}-{\text{r}}^{\prime }}{|{\text{c}}_{n}-{\text{r}}^{\prime }{|}^{3}}d^{3}{\text{r}}^{\prime }}}, \end{array}$$

respectively.

$S^{\infty }={\left(S_{ni}^{\infty }\right)}_{n,i}$ and $S^{corr,CG}={\left(S_{ni}^{corr,CG}\right)}_{n,i}$ are the secondary magnetic field integration matrices. Equations (34) and (35) can be rewritten into matrix equations,

(36) $$\begin{array}{l} {\text{B}}_{\infty ,h}^{s}=S^{\infty }{\text{u}}^{\infty }, \end{array}$$

and

(37) $$\begin{array}{l} {\text{B}}_{corr,h}^{s}=S^{corr,CG}{\text{u}}^{corr}, \end{array}$$

respectively.

An alternative treatment of ${\text{B}}_{corr}^{s}$ involves the already mentioned *conservation of charge property*, whose fulfillment is not guaranteed for the flux in Equation (31). Section 2.3.3 is dedicated to the description of this alternative.

#### 2.3.3. Conservative flux DG-FEM MEG forward problem

Following Equation (31), we can consider the analogous formula for the electric flux in the DG-FEM scheme, i.e.,

(38) $$\begin{array}{l} \sigma \nabla u_{h}^{corr}=\underset{i}{\sum }u_{i}^{corr}\nabla \varphi_{i}, \end{array}$$

where (φ_*i*_)_*i*_ is a basis of $V_{h}^{1}$.

As already mentioned, in general this discrete formulation of the flux does not verify the conservation of charge property. Conversely and despite the CG-FEM case, in the DG-FEM approach we can consider another expression of the discrete electric flux, i.e.,

(39) $$\begin{array}{l} {\text{j}}_{h}^{corr,DG}=\left\{\sigma \nabla u_{h}^{corr}\right\}-\eta \frac{{\hat{\sigma }}_{\gamma }}{h_{\gamma }}⟦u_{h}^{corr}⟧ \end{array}$$

that verifies the conservation of charge law, as described in Remark 3.

The main idea is to embed this *conservative current* (or *conservative flux*, i.e., flux fulfilling the property of conservation of charge) in the computation of ${\text{B}}_{corr}^{s}$. We have to notice that ${\text{j}}_{h}^{corr,DG}$ is defined only on the internal skeleton Γ_*int*_ (Equation 21) and not in the entire volume Ω. In order to integrate ${\text{j}}_{h}^{corr,DG}$ when computing ${\text{B}}_{corr}^{s}$ (Equation 28), we need to project the current into the volume. One way to do so is to interpolate ${\text{j}}_{h}^{corr,DG}$ in the space of the lowest-order Raviart Thomas function (*RT*_0_). *RT*_0_ is *H*(*div*)- conforming and its degrees of freedom (DOFs) are the evaluations of the basis functions along the projections normal to the faces of each element, exactly where ${\text{j}}_{h}^{corr,DG}$ is defined. The space *H*(*div*; Ω) is defined as:

(40) $$\begin{array}{l} H\left(div;\Omega \right):=\left\{\text{v}\in L^{2}{\left(\Omega \right)}^{3}:\nabla \cdot \text{v}\in L^{2}\left(\Omega \right)\right\}, \end{array}$$

and *RT*_0_ as (Nédélec, 1980; Fortin and Brezzi, 1991):

(41) $$RT_{0}(T_{h}(\Omega )):\text{​}=\{\text{v}\in H(div;\Omega ):(\nabla \cdot \text{v}){|}_{E}\in P^{0}(E),\forall E\in T_{h}(\Omega )\}.$$

As we are considering hexahedral elements, *P*^*l*^(*E*) in Equation (22) is ℚ^*l*^(*E*), defined as:

(42) $$\begin{array}{l} {\mathbb{Q}}^{l}\left(E\right)=\text{span}\left\{\Pi_{i=1}^{3}x_{i}^{\alpha_{i}}:\text{x}\in E,\alpha \in {\mathbb{N}}^{3},\text{max}\alpha_{i}\leq l\right\}, \end{array}$$

therefore also in Equation (41), we have *P*^0^ = ℚ^0^. For a regular, hexahedral mesh with edge length *h*, as in our case, a *RT*_0_ basis function **ψ**_*k*_ is supported on the two hexahedral elements $E_{e},E_{f}\in T_{h}\left(\Omega \right)$ sharing the face *f*_*k*_ = *E*_*e*_ ∩ *E*_*f*_ with normal vector **n**_*k*_ and centroid ${\bar{\text{x}}}_{k}$. It can be defined by

(43) $$\psi_{k}(\text{x})=\{\begin{array}{ll} \left(1+\frac{(\text{x}-{\bar{\text{x}}}_{k})\cdot {\text{n}}_{k}}{h}\right){\text{n}}_{k}, & \text{if }\text{x}\in {\bar{E}}_{e}\cap {\bar{E}}_{f}\\ 0, & \text{otherwise.} \end{array}$$

**Figure 1:**
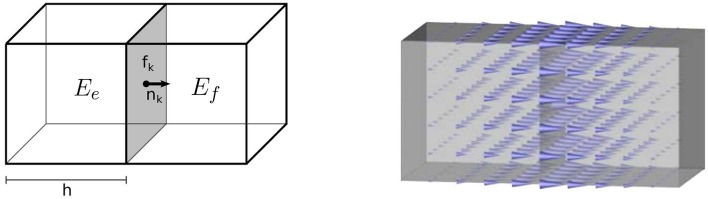
Visualization of a zeroth-order Raviart-Thomas basis function **(Right)** and its support **(Left)**. The support is made of two hexahedral elements *E*_*e*_ and *E*_*f*_, which are sharing the face *f*_*k*_ with unit outer normal **n**_*k*_. The vector valued function is equal to 1 · **n**_*k*_ on the face *f*_*k*_ and it decays when reaching the other parallel faces.

For more insights see Fortin and Brezzi (1991) and Nédélec (1980), and Figure 1, where the basis function **ψ**_*k*_ has been visualized.

For the discretization of ${\text{B}}_{corr}^{s}$ we can start from observing that the conservative flux $\Psi \left(u_{h}^{corr}\right)$ is a function of $L^{2}\left(\Gamma_{int}\right)$ which depends on the potential $u_{h}^{corr}$:

(44) $$\begin{array}{l} \text{Ψ}\left(u_{h}^{corr}\right)={\text{j}}_{h}^{corr,DG}=\left\{\sigma \nabla u_{h}^{corr}\right\}-\eta \frac{{\hat{\sigma }}_{\gamma }}{h_{\gamma }}⟦u_{h}^{corr}⟧\in L^{2}\left(\Gamma_{int}\right). \end{array}$$

If (φ_*i*_)_*i*_ is a basis of $V_{h}^{1}$, then the correction potential can be written as

(45) $$\begin{array}{l} u_{h}^{corr}=\underset{i}{\sum }u_{i}^{corr}\varphi_{i}, \end{array}$$

and, due to linearity, we have

(46) $$\begin{array}{l} \text{Ψ}\left(u_{h}^{corr}\right)=\underset{i}{\sum }u_{i}^{corr}\text{Ψ}\left(\varphi_{i}\right). \end{array}$$

If we now apply the projection Π_*RT*0_ into *RT*_0_ to $\Psi \left(u_{h}^{corr}\right)$ and we exploit again linearity, we obtain:

(47) $$\begin{array}{l} \Pi_{RT0}\left(\text{Ψ}\left(u_{h}^{corr}\right)\right)=\underset{i}{\sum }u_{i}^{corr}\Pi_{RT0}\left(\text{Ψ}\left(\varphi_{i}\right)\right)\in L^{2}\left(\Omega \right). \end{array}$$

Finally, ${\text{B}}_{corr}^{s}$ can be then approximated as follows:

(48) $$\begin{array}{l} {\text{B}}_{corr}^{s}\left(\text{r}\right)≃-\frac{\mu_{0}}{4\pi }\underset{i}{\sum }u_{i}^{corr}\int_{\Omega }\Pi_{RT0}\left(\text{Ψ}\left(\varphi_{i}\right)\right)\left({\text{r}}^{\prime }\right)\times \frac{\text{r}-{\text{r}}^{\prime }}{|\text{r}-{\text{r}}^{\prime }{|}^{3}}d^{3}{\text{r}}^{\prime }. \end{array}$$

If we call **c**_*n*_ the center of the *n*th coil, then the discretization of ${\text{B}}_{corr}^{s}$ evaluated in **c**_*n*_ reads,

(49) $$\begin{array}{l} {\text{B}}_{corr}^{s}\left({\text{c}}_{n}\right)≃\underset{i}{\sum }u_{i}^{corr}\underset{:=S_{ni}^{corr,DG}}{\underset{︸}{\left(-\frac{\mu_{0}}{4\pi }\right)\underset{\Omega }{\int }\Pi_{RT0}\left(\text{Ψ}\left(\varphi_{i}\right)\right)\left({\text{r}}^{\prime }\right)\times \frac{{\text{c}}_{n}-{\text{r}}^{\prime }}{|{\text{c}}_{n}-{\text{r}}^{\prime }{|}^{3}}d^{3}{\text{r}}^{\prime }}}. \end{array}$$

$S^{corr,DG}={\left(S_{ni}^{corr,DG}\right)}_{n,i}$ is the secondary magnetic field integration matrix related to the DG-FEM scheme. Equation (49) can be rewritten into a matrix equation,

(50) $$\begin{array}{l} {\text{B}}_{corr,h}^{s}=S^{corr,DG}{\text{u}}^{corr}, \end{array}$$

where ${\text{B}}_{corr,h}^{s}$ represents the discretization of ${\text{B}}_{corr}^{s}$.

**Remark 4**. *The projection of the *i*^*th*^ basis function of the space $V_{h}^{1}$ can be described as:*

(51) $$\begin{array}{l} \Pi_{RT_{0}}\left(\text{Ψ}\left(\varphi_{i}\right)\right)=\sum_{k}\alpha_{i}^{k}{\text{ψ}}_{k}, \end{array}$$

where (**ψ**_*k*_)_*k*_ form a basis of *RT*_0_ and $\alpha_{i}^{k}$ are the DOFs, which can be derived as

(52) $$\begin{array}{l} \alpha_{i}^{k}=\text{Ψ}\left(\varphi_{i}\left({\bar{\text{x}}}_{k}\right)\right)\cdot {\text{n}}_{k}, \end{array}$$

with ${\bar{\text{x}}}_{k}$ and **n**_*k*_ the centroid and the external normal of the face *f*_*k*_, respectively (see Figure 1).

### 2.4. Transfer matrix approach

As described in the next section, MEG forward computations will be carried out for a large number of dipole sources. In order to speed up the many numerically expensive computations of the secondary B-field **B**^*s*^ for all of these sources, following Wolters et al. (2004), we adapted and implemented transfer matrix approaches for all three presented FEM-based MEG forward modeling schemes.

If *K***u** = **j** represents the resulting linear system of the EEG forward computation discretization, we can formally write

(53) $$\begin{array}{l} \text{u}=K^{-1}\text{j}. \end{array}$$

If we combine Equations (53) and (50), we obtain

(54) $$\begin{array}{l} {\text{B}}_{corr,h}^{s}=S\text{u}=S\left(K^{-1}\text{j}\right)=\left(SK^{-1}\right)\text{j}=B_{MEG}\text{j}, \end{array}$$

where *S* is a generic secondary magnetic field integration matrix. *B*_*MEG*_ is the so-called MEG transfer matrix and allows computing ${\text{B}}_{corr,h}^{s}$ with a matrix-vector multiplication, instead of solving the EEG forward problem and applying *S*.

To compute $B_{MEG}=SK^{-1}$, we can multiply its definition by *K* from the right and then transpose it.

Using the symmetry of *K*, we arrive at the following matrix equation,

(55) $$\begin{array}{l} KB_{MEG}^{t}=S^{t}, \end{array}$$

which can be solved for each row of *S* (column of *S*^*t*^).

## 3. Methods

### 3.1. Implementation

We implemented the CG-FEM and the two DG-FEM approaches [non-conservative Equation 38 and conservative flux (Equation 39)] for the MEG forward problem in the Distributed and Unified Numerics Environment (DUNE)^1^ (Blatt and Bastian, 2007; Bastian et al., 2008a,b). DUNE is a modular open source C++ library for solving partial differential equations with mesh-based methods. In particular, we used the DUNE-ALUGrid module (Alkämper et al., 2016) for the representation of hexahedral meshes and the DUNE-PDELab module (Bastian et al., 2010) for the discretization of the partial differential equations. All the newest implementations have been gathered in the module called DUNEuro^2^, a special module dedicated to solve PDEs in neuroscience.

### 3.2. Volume conductor models

For numerical accuracy tests of our new CG- and DG-FEM implementations, we generated 4-layer homogeneous sphere models for which an analytical solution for the MEG exists (see section 2.1.4). We used four compartments with different conductivities in order to evaluate if, besides the analytical solution in Equation (15), also our numerical implementations show conductivity-independence of MEG in spherical volume conductors and because the four compartment model is closer to a realistic head model as shown in **Figure 10**. The four compartments, whose radii and conductivities are shown in Table 1 (same parametrization as in Engwer et al., 2017), are rough approximations for skin, skull, cerebrospinal fluid (CSF) and brain compartments. The spherical domain is represented via hexahedral meshes with three different resolutions, namely 4, 2, and 1 mm. In this work, we focused on hexahedral meshes in order to study the scenario where the combination of thin skull structures and insufficient hexahedral mesh resolutions might result in so-called skull leakages, as in Engwer et al. (2017). The numbers of vertices and elements of these meshes are shown in Table 2.

**Table 1:** Four compartment sphere model.

**Tissue**	**Outer radius (mm)**	**Conductivity(S/m)**	**References**
Brain	78	0.33	Ramon et al., 2004
CSF	80	1.79	Baumann et al., 1997
Skull	86	0.01	Dannhauer et al., 2011
Skin	92	0.43	Ramon et al., 2004; Dannhauer et al., 2011

**Table 2:** Parameters (from left to right) of the regular hexahedral meshes of the 4-layer sphere models used for validation purposes: segmentation resolution (Segm. Res.), mesh width (h), number of vertices and number of elements.

	**Segm. Res. (mm)**	**Mesh width (h)(mm)**	**No. of vertices**	**No. of elements**
*seg*_4_*res*_4	4	4	56,235	51,104
*seg*_2_*res*_2	2	2	428,185	407,907
*seg*_1_*res*_1	1	1	3,342,701	3,262,312

### 3.3. Sources and sensors

As only tangential orientation components produce an MEG signal in a multi-layer sphere model (section 2.1.4), we generated 8,000 dipoles with purely tangential orientations and unit strengths. The sources were uniformly distributed inside the brain compartment on spherical surfaces with 8 different logarithmically scaled eccentricities reported in Table 3. A source with eccentricity value of 0 is positioned in the center of the sphere, while a source with eccentricity value of 1 belongs to the surface separating brain and CSF compartments. The logarithmic scaling was chosen, since it is well known that numerical errors of the subtraction approach increase with decreasing distance of a source to the next conductivity jump (Wolters et al., 2007; Drechsler et al., 2009). We therefore expect larger numerical errors especially for the sources at the highest eccentricity of 0.9873, which only have a distance of 0.99 mm to the CSF compartment.

**Table 3:** Source eccentricities and corresponding distances to the CSF compartment.

**Eccentricity**	**0.1**	**0.5025**	**0.7487**	**0.8718**	**0.9334**	**0.9642**	**0.9796**	**0.9873**
Distance to CSF comp. (mm)	77.22	38.80	19.60	9.99	5.19	2.79	1.59	0.99

As the cortex has a thickness of 4 to 2 mm (Hämäläinen et al., 1993; Murakami and Okada, 2006) and the sources are located in the center of the gray matter, the sources which are most important to analyze are those with a distance of 2 to 1 mm to the CSF compartment. Therefore we will focus on the results of sources whose eccentricities are between 0.9642 (2.79 mm from the CSF compartment) and 0.9873 (0.99 mm from the CSF compartment) and especially on those with the middle value of this range, i.e., 0.9796 (1.59 mm from the CSF compartment). Furthermore, *in praxis* (and for the realistic head model used in this study, section 3.6), sources are usually placed so that at least one layer of elements is between the source element and the conductivity jump, which is fulfilled for the considered eccentricities ⩽ 0.9873 in the 1 mm model (*seg*_1_*res*_1) and ⩽ 0.9642 in the 2 mm model (*seg*_2_*res*_2). See Table 3 for details on the eccentricities and the corresponding distance from the CSF compartment.

**Figure 2:**
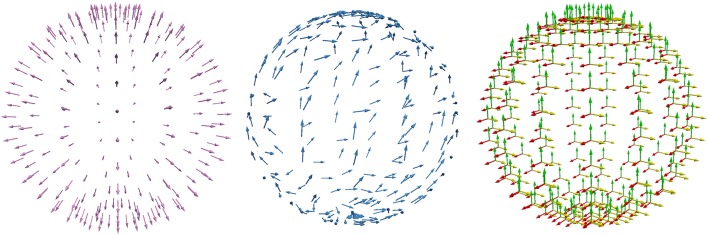
Visualization of the 256 point-magnetometers used in the sphere model analysis. Radially **(Left)** and tangentially **(Middle)** oriented point-magnetometers have been employed exclusively in section 4.1.1, while in all other studies all the three Cartesian components **(Right)** of the vector fields **B**^p^, **B**^s^, *and* **B** *have been considered*.

With regard to the MEG sensors, we used 256 point-magnetometers outside the sphere model at a fixed radius of 110 mm (see Figure 2).

### 3.4. Error measures

We will use the two error metrics that are commonly used for validating EEG and MEG forward approaches (Meijs et al., 1989; Bertrand et al., 1991; Marin et al., 1998; van den Broek et al., 1998; Schimpf et al., 2002; Wolters et al., 2007), namely, the relative difference measure (RDM%) for topographical errors:

(56) $$\begin{array}{l} RDM\%\left({\text{f}}_{ana},{\text{f}}_{num}\right)=50{\left\|\frac{{\text{f}}_{num}}{||{\text{f}}_{num}|{|}_{2}}-\frac{{\text{f}}_{ana}}{||{\text{f}}_{ana}|{|}_{2}}\right\|}_{2}, \end{array}$$

and magnitude error (MAG%):

(57) $$\begin{array}{l} MAG\%\left({\text{f}}_{ana},{\text{f}}_{num}\right)=100\left(\frac{||{\text{f}}_{num}|{|}_{2}}{||{\text{f}}_{ana}|{|}_{2}}-1\right), \end{array}$$

where **f** is either the secondary B-field **B**^*s*^ or the full B-field **B**. Note that we considered vector-magnetic fields (**B**^*p*^, **B**^*s*^, **B**) without projecting them into radial nor tangential directions, i.e., without distinguishing between radial and tangential point-magnetometers.

Statistical results of numerical accuracies will be visualized with mean curves and boxplots (see **Figures 5–9**). In the boxplots, the analysis includes maximum and minimum, indicated by upper and lower error bars, and thereby the total range (TR). Furthermore, it includes the interval between upper and lower quartile, i.e., the interquartile range (IQR), which is marked by a box with a black dash showing the median.

### 3.5. A leaky model

The study of Engwer et al. (2017) showed for EEG forward scenarios that DG-FEM can considerably outperform CG-FEM in skull leakage models, where the sphere model is discretized with a hexahedral mesh of 2 mm resolution and where at the same time the thickness of the skull compartment is deliberately reduced down to 2 mm (*seg*_2_*res*_2_*r*82), so that it contains many *leaky points*, i.e., vertices belonging to both an element labeled as skin and an element labeled as CSF or brain. See Table 4 for details. Note that real skull holes are not investigated in this study. The sources are the same as previously described. Even when not expecting similarly substantial error reductions on the MEG side, we used here the same leakage models as in Engwer et al. (2017) to investigate the influence of skull leakages on the presented CG- and DG-FEM MEG approaches.

**Table 4:** Parameters (from left to right) of the regular hexahedral meshes of the 4-layer sphere models used to investigate the influence of skull leakages on the presented CG- and DG-FEM MEG approaches: segmentation resolution (Segm. Res.), mesh width (h), outer radius of the skull (mm) and number of leaky points.

	**Segm. Res. (mm)**	**Mesh width (h)(mm)**	**Outer skull radius(mm)**	**No. of leaky points(mm)**
*seg*_2_*res*_2	2	2	86	0
*seg*_2_*res*_2_*r*82	2	2	82	10,080

### 3.6. A realistic head model

As a proof of concept, we computed one MEG forward solution using the DG-FEM approach in a more realistic scenario. Based on MRI recordings of a human head, a segmentation considering six tissue compartments (white matter, gray matter, cerebrospinal fluid, skull compacta, skull spongiosa, and skin) that includes realistic skull openings such as the foramen magnum and the optic nerve canal was generated. Based on this segmentation, a six-compartment realistically shaped head model was built, a hexahedral mesh of 2 mm resolution resulting in 508,412 vertices and 484,532 elements (**Figure 10**). As this model was not corrected for leakages, 1,164 vertices belonging to both CSF and skin elements were found. These leaky points were mainly located at the temporal bone. More details about the model and its generation process can be found in Engwer et al. (2017). Locations and orientations of the sensors were chosen accordingly to the CTF machine (OMEGA2005, CTF, VSM MedTech Ltd., Canada), see **Figure 10**.

## 4. Results

In this section, the results relative to the evaluation and validation in multi-layer homogeneous sphere will be presented, followed by the results of one forward computation on a realistically shaped head model.

### 4.1. Validations and evaluations in spherical volume conductor models

In this section, we will validate, compare and evaluate the three developed and implemented approaches for the MEG forward problem, namely the CG-FEM and the DG-FEM with non-conservative (Equations 31, 38) and conservative flux (Equation 39), in spherical volume conductor models.

#### 4.1.1. Preparatory work using analytical approach

To recall the most important symmetry properties of the MEG forward problem in spherical volume conductor models, to prepare the numerical studies below and to enable an easier interpretation of their results, we first tested and visualized the properties of the MEG analytical solution for a multi-layer homogeneous sphere model, as reported in Remark 1. Here, we consider radial and tangential point-magnetometers, i.e., we have projected the B-field (**B**^*p*^,**B**^*s*^,**B**) onto the radial **n** and tangential **t** directions at sensor locations (Figure 2).

**Figure 3:**
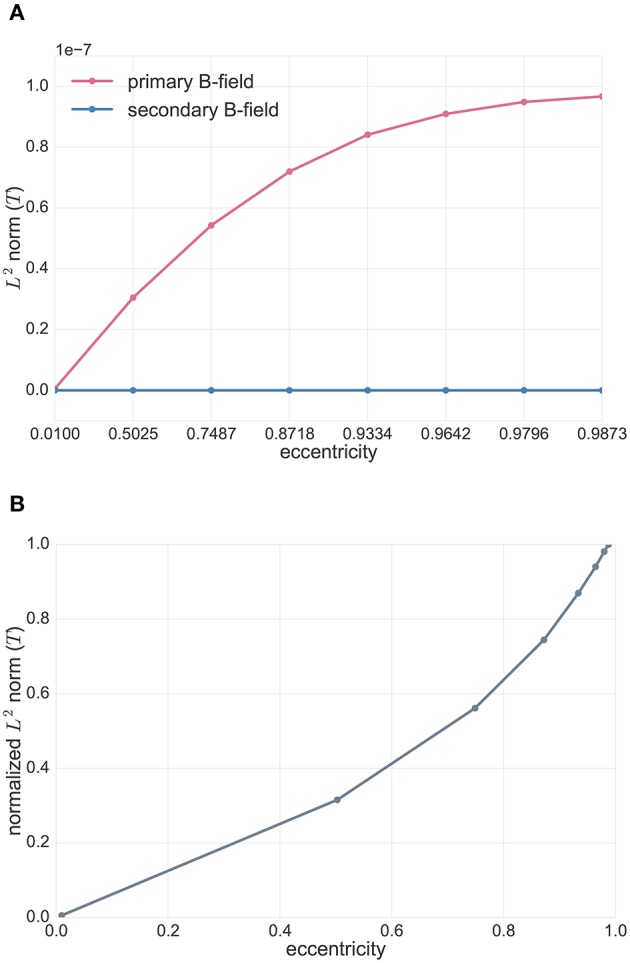
**(A)** Analytical solutions in spherical volume conductor model for radial point-magnetometers: *L*^2^ norm of the primary (**B**^*p*^, pink) and secondary (**B**^*s*^, blue) B-fields (see Equation 58) for tangentially-oriented sources at logarithmically scaled eccentricities. Values are expressed in Tesla *T*. **(B)** Analytical solutions in spherical volume conductor model for radial point-magnetometers: *L*^2^ norm of the radial full B-field component relative to the one for the most eccentric source (see Equation 59) for tangentially-oriented sources at logarithmically scaled eccentricities.

In Figure 3A, we compared, for the tangentially-oriented sources at logarithmically scaled eccentricities and the 256 radial point-magnetometers, the *L*^2^ norm of primary **B**^*p*^ (in pink) and secondary **B**^*s*^ (in blue) B-fields, i.e.,

(58) $$\begin{array}{l} ||{\text{B}}^{p}\cdot \text{n}|{|}_{2},||{\text{B}}^{s}\cdot \text{n}|{|}_{2}. \end{array}$$

We notice that the only contribution to radial point-magnetometers is given by the primary component of the B-field, **B**^*s*^, as proven in Sarvas (1987).

In Figure 3B, we plotted the *L*^2^ norm of the full B-field for radial point-magnetometers normalized to the maximum over all tested sources, which is achieved for the most eccentric source, i.e.,

(59) $$\begin{array}{l} \frac{||\text{B}\cdot \text{n}|{|}_{2}}{\text{max}||\text{B}\cdot \text{n}|{|}_{2}}. \end{array}$$

We can see how the magnitude of the full B-field increases for sources with an increasing eccentricity.

**Figure 4:**
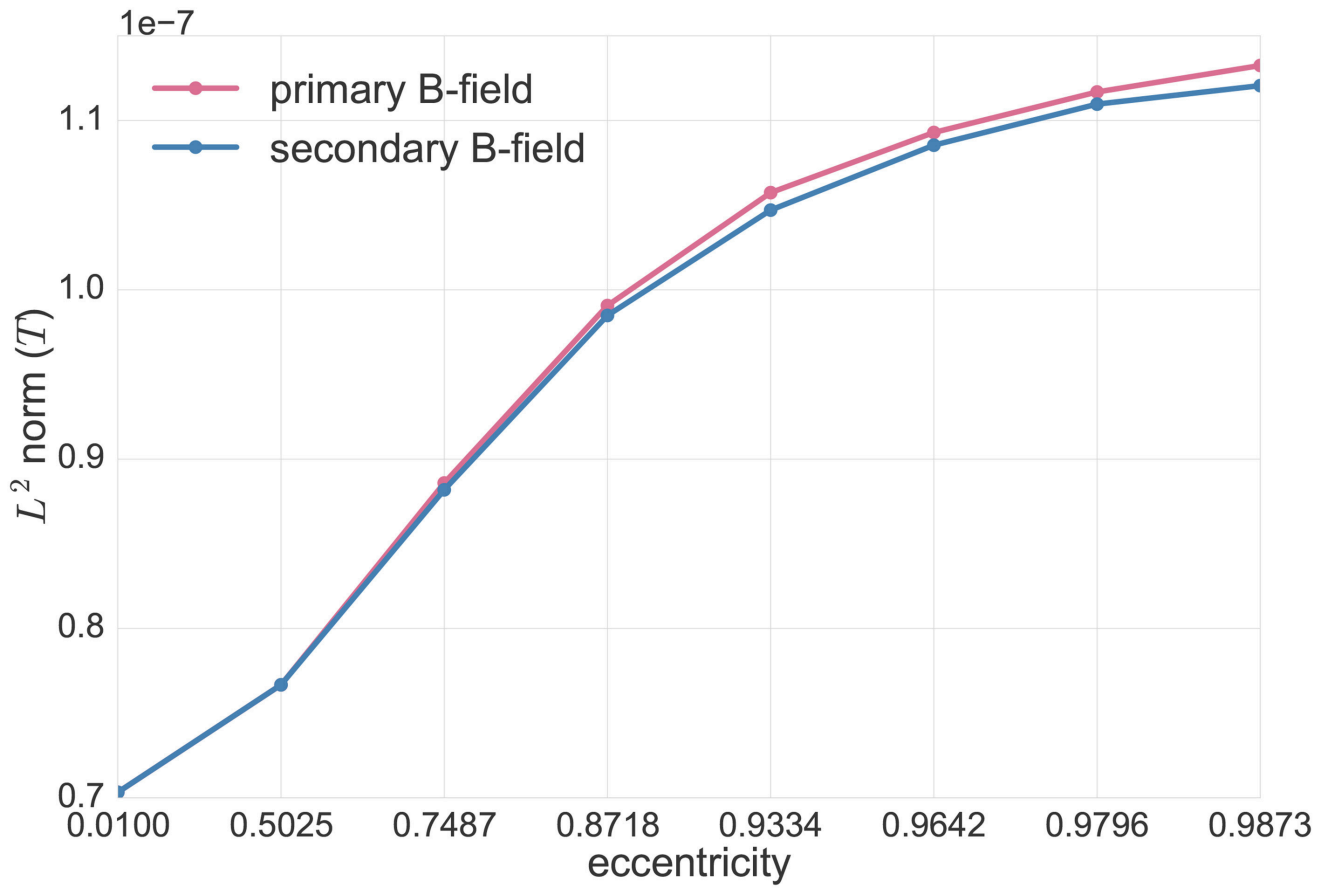
Analytical solutions in spherical volume conductor model for tangential point-magnetometers: *L*^2^ norm of the primary (**B**^*p*^, pink) and secondary (**B**^*s*^, blue) B-fields (see Equation 60) for tangentially-oriented sources at logarithmically scaled eccentricities. Values are expressed in Tesla *T*.

In Figure 4, we investigated the analytical solutions in the spherical volume conductor model for tangential point-magnetometers (Figure 2, middle). The Figure shows the *L*^2^ norm of the primary (in pink) and secondary (in blue) tangential B-field components, i.e.,

(60) $$\begin{array}{l} ||{\text{B}}^{p}\cdot \text{t}|{|}_{2},||{\text{B}}^{s}\cdot \text{t}|{|}_{2}, \end{array}$$

for tangentially-oriented sources at different eccentricities.

In this Figure we can see that, for tangential point-magnetometers, the deeper the sources are, the more the primary and secondary B-fields give identical contributions, but with opposite signs, to the full B-field, i.e., they more and more cancel each other out. Toward the sphere center, sources become more and more radial and the full B-field goes down to zero. However, as Figure 4 also shows, with increasing source eccentricity the relative contribution of the primary tangential B-field component increases when compared to the secondary B-field component. The tangential full B-field projection (i.e., **B** · **t**) and, together with it, the difference between primary and secondary tangential B-field components (i.e., **B**^*p*^ · **t** and **B**^*s*^ · **t**) thus increase with increasing source eccentricity.

#### 4.1.2. FEM study 1: conservative vs. non-conservative flux for DG approach

**Figure 5:**
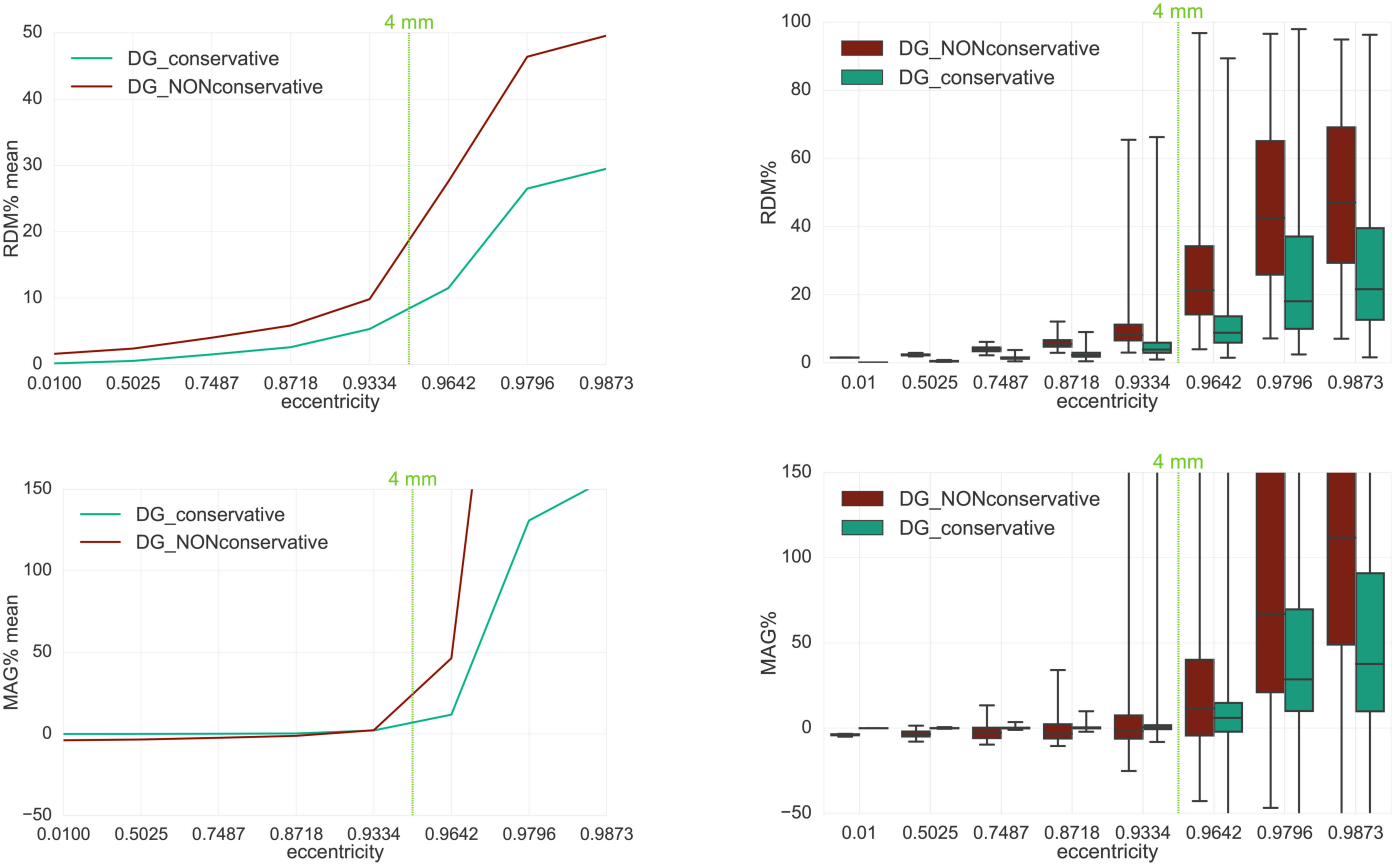
Accuracy comparison for secondary B-field **B**^*s*^ computation (Equation 28) between DG-FEM with non-conservative flux (Equation 38, in red) and DG-FEM with the conservative flux (Equation 39, in green) in a 4 mm hexahedral sphere model: visualized are the means **(Left column)** and the boxplots **(Right column)** of the RDM% **(Top row)** and MAG% **(Bottom row)**, for tangentially oriented sources at logarithmically-scaled eccentricities. Dipoles not belonging to the brain compartment are excluded from the statistics. The dashed green line represents the eccentricity of 4 mm distance to the brain-CSF boundary. Note the different scaling of the y-axes **(Top row)**.

**Figure 6:**
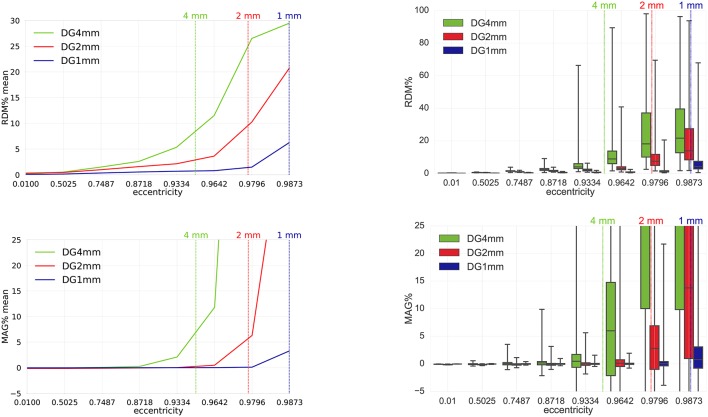
Validation and convergence analysis for secondary B-field **B**^*s*^ computation (Equation 28) of DG-FEM with conservative flux (Equation 39) in a 4 mm (green), 2 mm (red) and 1 mm (blue) hexahedral sphere model: visualized are the means **(Left column)** and the boxplots **(Right column)** of the RDM% **(Top row)** and MAG% **(Bottom row)**, for tangentially oriented sources at logarithmically-scaled eccentricities. Dipoles not belonging to the brain compartment are excluded from the statistics. Dashed lines represent the eccentricities of 4 mm (green), 2 mm (red) and 1 mm (blue) distances to the brain-CSF boundary. Note the different scaling of the y-axes **(Top row)**.

**Figure 7:**
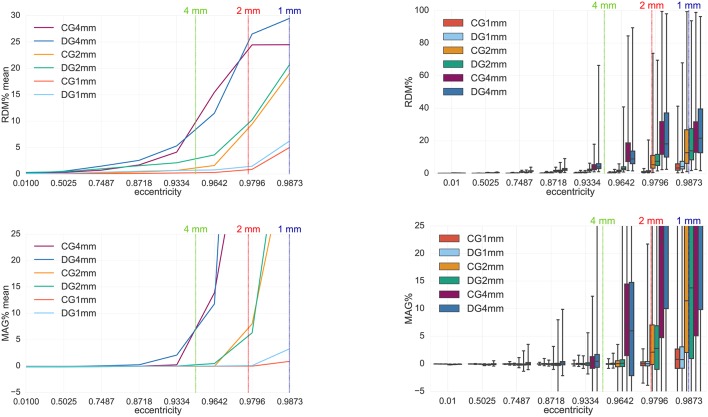
Accuracy comparison for secondary B-field **B**^*s*^ computation (Equation 28) between CG-FEM (in warm colors) and DG-FEM with the conservative flux (in cold colors), for different mesh resolutions: visualized are the means **(Left column)** and the boxplots **(Right column)** of the RDM% **(Top row)** and MAG% **(Bottom row)**, for tangentially oriented sources at logarithmically-scaled eccentricities. Dipoles not belonging to the brain compartment are excluded from the statistics. Dashed lines represent the eccentricities of 4 mm (green), 2 mm (red) and 1 mm (blue) distances to the brain-CSF boundary. Note the different scaling of the y-axes **(Top row)**.

**Figure 8:**
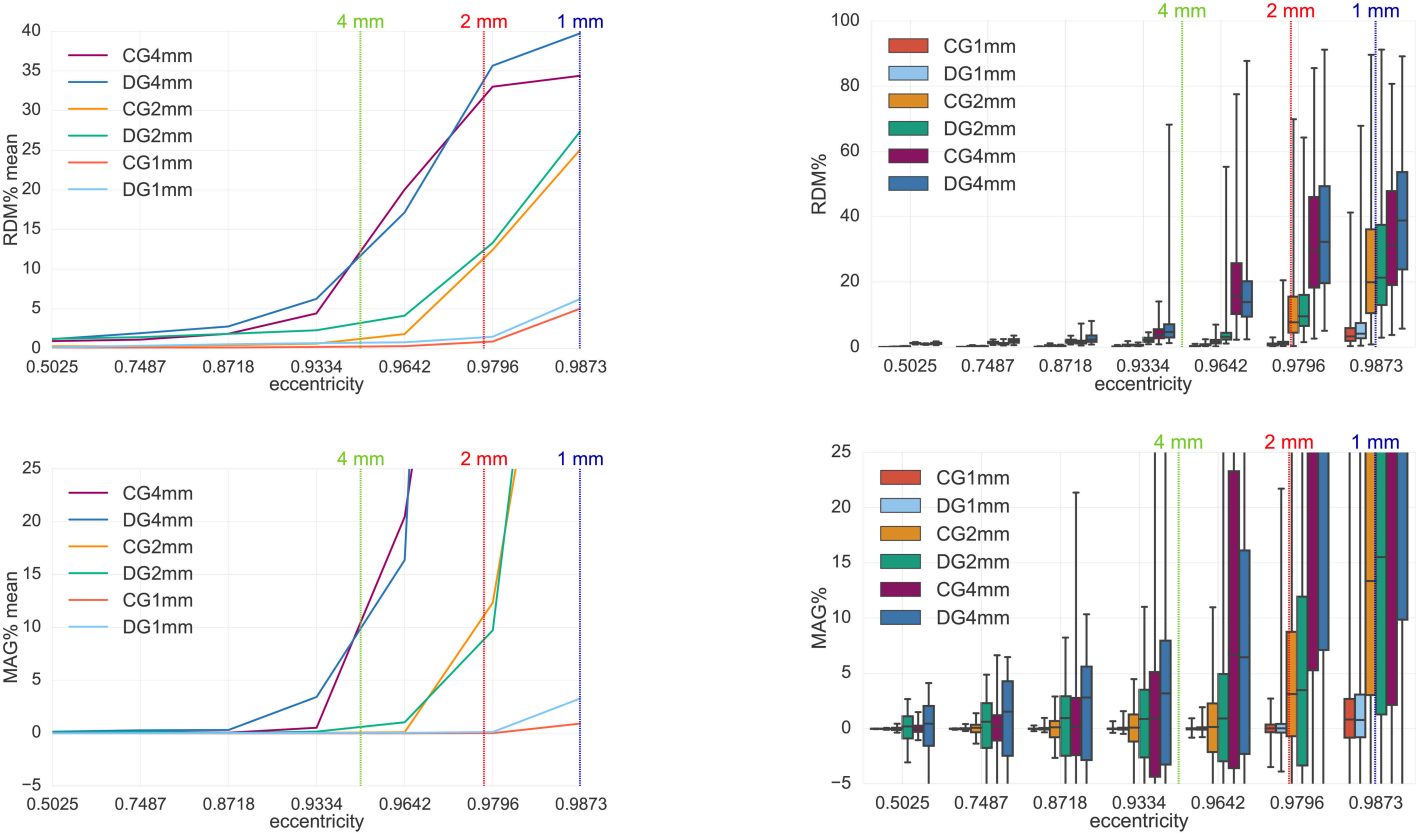
Accuracy comparison between CG- and DG-FEM for solving the MEG forward problem, i.e., the full B-field **B** (Equation 14), for different mesh resolutions. Visualized are the means **(Left column)** and the boxplots **(Right column)** of the RDM% **(Top row)** and MAG% **(Bottom row)**, for tangentially oriented sources at logarithmically-scaled eccentricities. Dipoles not belonging to the brain compartment are excluded from the statistics. Dashed lines represent the eccentricities of 4 mm (green), 2 mm (red) and 1 mm (blue) distances to the brain-CSF boundary. Note the different scaling of the y-axes **(Top row)**.

**Figure 9:**
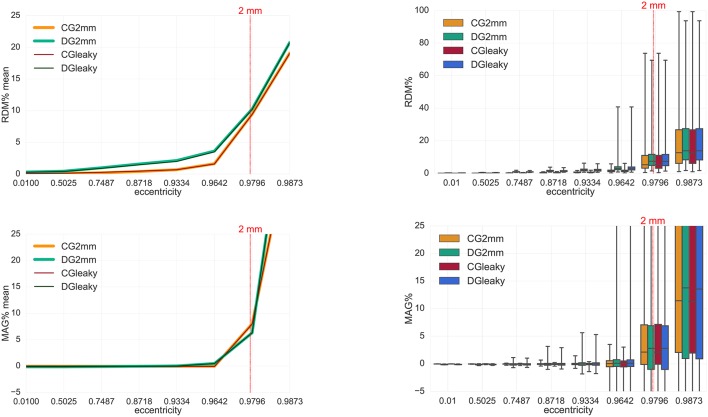
Accuracy comparison for secondary B-field **B**^*s*^ computation (Equation 28) between CG-FEM (in warm colors) and DG-FEM with the conservative flux (in cold colors), in two different 2 mm hexahedral sphere models: *seg*_2_*res*_2 and *seg*_2_*res*_2_*r*82, described in Table 4. Visualized are the means **(Left column)** and the boxplots **(Right column)** of the RDM% **(Top row)** and MAG% **(Bottom row)**, for tangentially oriented sources at logarithmically-scaled eccentricities. Dipoles not belonging to the brain compartment are excluded from the statistics. The dashed red line represents the eccentricity of 2 mm distance to the brain-CSF boundary. Note the different scaling of the y-axes **(Top row)**.

**Figure 10:**
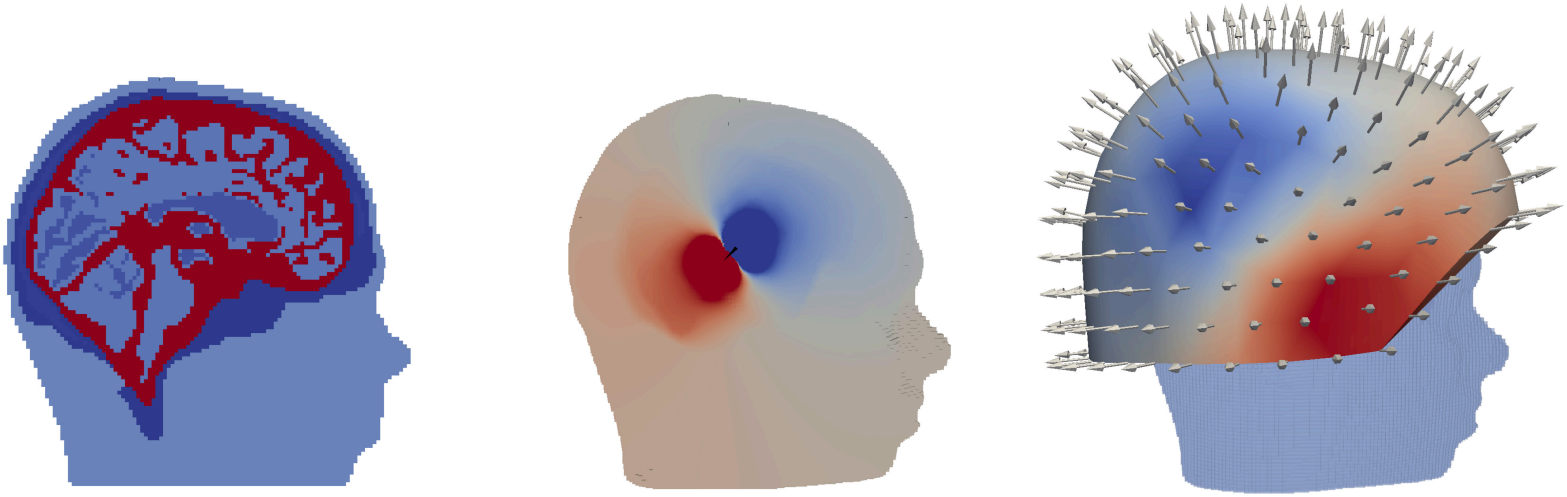
Exemplary EEG and MEG forward computation for an auditory source computed using DG-FEM in a realistically shaped head model. Hexahedral mesh with 2 mm resolution, 6 compartments, sagittal slice **(Left)**; electric potential distribution visualized on the clipped volume conductor model in the sagittal plane where the auditory dipole (black cone) lies **(Middle)**; MEG solution interpolated on the radial magnetometers including a volume rendering of the head model **(Right)**.

We now turn our interest to the validation and evaluation of our implemented new numerical FEM approaches for the MEG forward problem in spherical models. We will only consider tangentially-oriented sources for the validations and evaluations in the next sections, because, as seen in section 4.1.1, radial sources do not produce any magnetic field outside spherical volume conductor models. Following Equations (28) and (14), we will from now on measure errors of the vector fields **B**^*s*^ (Figures 5, 6, 7, **9**) and **B** (Figure 8). These errors thus include parts from the radial and the two tangential sensor orientations and thus enable an overall view on the MEG forward modeling accuracy. On the one hand, radially-oriented sensor orientations are dominant in realistic MEG sensor configurations (see **Figure 10**), while on the other hand, and as seen in section 4.1.1, because of the cancellation effect of primary and secondary B-fields, tangentially-oriented sensor orientations are especially delicate numerical test-cases.

In this analysis the focus is on DG-FEM and the necessity of embedding the conservative flux (Equation 38) in the evaluation of the secondary B-field **B**^*s*^. We will thus validate and compare the DG-FEM MEG forward methods with non-conservative (Equation 38) and conservative (Equation 39) flux.

The RDM% and MAG% statistical errors can be seen in Figure 5. Note that only a 4 mm mesh (*seg*_4_*res*_4) has been used, because the lower the resolution, the higher we expect the difference to be. With increasing source eccentricity, an overall increase of the RDM% (top row) and MAG% (bottom row) errors can be observed as shown by the mean error (left column) and by the boxplot statistics (right column). The boxplots indicate mainly increasing error statistics with regard to median, total range (TR), interquartile range (IQR) and also maxima for both conservative and non-conservative flux implementations. As a general result, the employment of the conservative flux (in green) delivers better results than the one of the non-conservative flux (in dark red). The difference between the two implementations is more evident with increasing eccentricity of the sources.

Let us now discuss in more detail the eccentricity of 0.9796, i.e., 1.59 mm from the brain-CSF boundary. Higher eccentricities do not have practical importance, as already explained in section 3.3. For the eccentricity of 0.9796, the maximum difference of 20 *percentage points* (pp) in mean RDM% is achieved between the conservative and the non-conservative DG flux approaches. For the least eccentric sources, this difference goes down to about 2 pp (see the 0.01 eccentricity in top left subfigure of Figure 5).

With regard to the boxplot of the RDM%, the median values of the conservative flux case are overall smaller than the ones of the non-conservative flux. For sources with eccentricity value of 0.9796 the RDM% median difference is greater than 20 pp; the IQR difference is approximately 15 pp and the TR is constant and similar for both approaches.

In the MAG% boxplot (right column), the much better performance of the conservative flux approach is especially clearly visible. The MAG% median difference reaches 40 pp for realistic sources of eccentricity 0.9796. For the same sources, the TRs, IQRs and means are in general large, with a ratio 1:4 between conservative and non-conservative flux values. For lower eccentricities, we observe overall smaller errors.

#### 4.1.3. FEM study 2: convergence of DG approach

Since we have seen in the last study that the conservative flux DG-FEM approach (Equation 39) performs remarkably better than the non-conservative approach (Equation 38), for the remainder of the paper, we proceed with DG-FEM as in Equation (49). The third study proposed is about the convergence of the DG-FEM for computing the secondary B-field **B**^*s*^, when the mesh resolution is increased, namely from the coarsest resolution of 4 mm over 2 mm to the highest resolution of 1 mm. We studied the behavior of the RDM% and MAG% errors for 8,000 tangentially oriented and randomly distributed dipoles at different eccentricities. Results can be seen in Figure 6.

The RDM% and MAG% error mean curves (Figure 6, left column) are overall increasing with increasing source eccentricity, as hypothesized by the theory of the subtraction approach (Wolters et al., 2007) and well-known already from EEG results (Drechsler et al., 2009). Most importantly, for increasing mesh resolution, error statistics improve considerably. For the most relevant eccentricity of 0.9796, the highest resolved model (*seg*_1_*res*_1) reaches mean RDM% and MAG% errors of 1.5% and 0.1%, respectively. On the right column, we can study the boxplots of the RDM% and MAG% of the same scenario analyzed before. Both in the RDM% and MAG% cases, there is an overall increase of the median, TR and IQR when increasing the source eccentricity and decreasing the mesh resolution. If we focus on the 1 mm mesh and 0.9796 eccentricity, the RDM% median is only around 1.2%; the IQR is 0.8% and the TR reaches 20%. In particular, the IQR for dipoles of eccentricity 0.9796 increases drastically from 0.8% (1 mm) to almost 10% (2 mm) and 30% (4 mm). The TR behaves similarly. The median MAG% is extremely low, i.e., ≈0.017%; the IQR is ≈0.8% and the TR is ≈25%. For this eccentricity, we notice a huge difference among the three mesh resolutions: the medians grow from 0.017% (1 mm), to 2.8% (2 mm), up to 28.6% (4 mm). The same trend is noticeable for the IQR: 0.8% (1 mm), 7% (2 mm) and 60% (4 mm). However, these values are out of the displayed graph range. The TR again behaves similarly.

#### 4.1.4. FEM study 3: comparison between CG and DG

The fourth analysis performed in this work is a comparison between CG- and DG-FEM for the MEG forward computation. RDM% and MAG%s are evaluated both for the secondary B-field **B**^*s*^ (Figure 7) and the full B-field **B** (Figure 8), following Equations (28) and (14), respectively.

In our following result discussion, we focus on the comparison between the two methods, rather than the performance of each method alone, which has been done for DG-FEM in section 4.1.3.

With regard to the secondary B-field **B**^*s*^ results, we will first analyze the mean RDM% curve (Figure 7, top left). In this plot we can distinguish the three different couples of curves: CG- and DG-FEM for 1 mm (*seg*_1_*res*_1), 2 mm (*seg*_2_*res*_2) and 4 mm (*seg*_4_*res*_4).

If we focus on the 1 mm analysis, we notice a high accuracy (up to around 1.5%) for eccentricities smaller or equal to 0.9796 (i.e., 1.59 mm from the CSF compartment). Even if in our current implementation, CG-FEM achieves slightly better results, the differences to DG-FEM are below 0.5 pp, so that in summary, DG-FEM constitutes an interesting alternative to the CG-FEM approach. Also for lower mesh resolutions of 2 and 4 mm, the performance of CG- and DG-FEM are very comparable for the realistic eccentricities up to 0.9796. A similar observation can be made for the mean MAG% curve, as the general trend for the three couples of curves (i.e., CG-DG 1 mm, CG-DG 2 mm, CG-DG 4 mm) is the same as before. When focusing on sources with eccentricity value of 0.9796, the mean MAG% difference between CG- and DG-FEM remains below 0.11 pp.

As for the boxplots for 1 mm mesh resolution (*seg*_1_*res*_1) and source eccentricity of 0.9796, the median RDM% difference is ≈0.4 pp (≈0.8 and ≈1.2% for CG- and DG-FEM, respectively); the IQR difference is around 0.2 pp (≈0.6 and ≈0.8% for CG- and DG-FEM, respectively) and the TR difference reaches almost 20 pp (Figure 7, top right). In the same scenario, the MAG% medians are identically extremely low, i.e., ≈0.015%. The IQRs also do not differ, while, again the TR difference is around 20 pp (Figure 7, bottom right).

The results when focusing on the full B-field **B** in Figure 8 are similar to the ones in Figure 7. Even for the full B-field, both the CG- and DG-FEM show an overall very high accuracy and a negligible difference, especially when focusing on the 1 mm study and source eccentricity of 0.9796. The mean RDM% (Figure 8, top left) is ≈0.9% for CG-FEM and ≈1.5% for DG-FEM; the mean MAG% is ≈ −0.015% for CG-FEM and ≈0.1% for DG-FEM (Figure 8, bottom right). With regard to the RDM% boxplot (Figure 8, top right), the medians are ≈0.8 and ≈1.15% for CG- and DG-FEM, respectively; the IQRs are ≈1 and ≈1.2% for CG- and DG-FEM, respectively, and the TRs are ≈3 and ≈20% for CG- and DG-FEM. In the MAG% boxplot (Figure 8, bottom right), we observe identical and extremely low values for the median (≈0.01%) and for the IQRs (≈0.8%). The difference of TRs is again bigger (≈20 pp) because of few outliers. Note that in Figure 8, we have omitted the errors for the lowest eccentricity of 0.01 because radial sources do not produce any magnetic field.

#### 4.1.5. FEM study 4: CG and DG in a leaky sphere model

Motivated by the EEG results of Engwer et al. (2017), where DG-FEM could clearly outperform CG-FEM in skull leakage scenarios, this section is concerned with the comparison of CG- and DG-FEM for the same scenario, but for the MEG case. Therefore, a *leaky sphere model* (*seg*_2_*res*_2_*r*82) has been constructed using an outer skull radius of 82 mm (instead of 86 mm as in the previous sections), resulting in an only 2 mm thick spherical skull compartment. Then, a 2 mm resolution hexahedral model has been constructed, resulting in 10,080 skull leakages. Again, only tangentially-oriented dipoles have been examined.

In Figure 9, we computed RDM% and MAG% mean curves (left column) and boxplots (right column) for the leaky skull spherical model scenario (*seg*_2_*res*_2_*r*82) compared to the *non-leaky* skull spherical model scenario (*seg*_2_*res*_2).

We observe that, in contrast to the improvement that DG-FEM could achieve in the EEG case (Engwer et al., 2017), the skull leakages do not visibly influence the numerical simulation of the secondary B-field **B**^*s*^ and, since the primary B-field **B**^*p*^ is also not influenced, thereby also the full B-field and thus the MEG forward problem.

If we observe the plots in the left columns, we notice that the curves of the leaky scenarios are completely overlaying the curves of the non-leaky scenarios, both for CG- and DG-FEM and both for RDM% and MAG% mean curves.

Also in the boxplots we cannot distinguish the behavior of the RDM% and MAG% in the leaky or non-leaky scenarios.

### 4.2. DG-FEM MEG study in a realistic head model

In the last study, as a proof of concept, we simulated an auditory N1 MEG signal using the new DG-FEM method with conservative flux (Equation 39) in the 6 compartment realistically-shaped head volume conductor model. Following experimental evidence (Okamoto et al., 2007), the N1 current dipole was positioned in the secondary auditory cortex and oriented inwards-pointing and normally to the gray matter surface. The result is shown in Figure 10. The subfigure on the left represents a sagittal slice through the head model, color-coding the 6 tissue compartment with different conductivities. In the middle and right subfigures, the results for EEG and MEG forward problem are presented. More precisely, the dipolar electrical potential map with frontal negativity and right occipital positivity is visualized on a cropped volume of the hexahedral mesh together with the underlying source (black arrow). The normally-oriented B-field MEG results at the 275 magnetometers was interpolated and visualized, showing a dipolar pattern that is 90° rotated to the EEG one and, following the right-hand rule, the negativity (blue) is over central and the positivity over temporal areas, in line with the experimental results (Hämäläinen et al., 1993; Okamoto et al., 2007).

## 5. Discussion

In this paper, we developed, implemented and evaluated one CG-FEM and two new DG-FEM approaches, a conservative and a non-conservative one, to solve the MEG forward problem. In section 2, we provided the mathematical theory for the CG-FEM and for the two new DG-FEM approaches with conservative and non-conservative discrete representation of the electrical flux. We started from the EEG formulation and continued with the MEG approaches. In section 3, we first described the implementation of the FEM-based MEG forward approaches in DUNEuro, a new modular C++ toolbox dedicated to solve partial differential equations in neuroscience. Furthermore, we presented the validation and evaluation platform for the new methods. In section 4, we presented the results of our analysis.

First, we tested and visualized the symmetry properties of the MEG analytical solution for a multi-layer homogeneous sphere model, as described in Remark 1 and as proven by Sarvas (1987). First of all, radial sources have a zero magnetic field outside a multi-layer sphere volume conductor model. Then, for tangential sources, only the primary B-field contributes to the full B-field for radial point-magnetometers (Figure 3A). For tangential sources and tangential point-magnetometers, we additionally showed that the more eccentric the source is, the more its primary B-field contributes to the full B-field relative to the contribution of the secondary B-field. The deeper the tangential source is, the more the secondary B-field weakens the primary B-field until for sources in the middle of the sphere model, where the primary and secondary B-fields totally compensate for each other (Figure 4 together with Figure 3B). In contrast, the more eccentric the sources are, the less symmetric the return currents are and the less their secondary B-field compensates the magnetic field of the primary current (Figure 4). This is in line with the fact that the strength of the full B-field for both radial and tangential magnetometers is decreasing for decreasing eccentricities (Figure 3B), as expected by the theory where it is proven that MEG sensors are blind for radial sources (Sarvas, 1987).

In a second analysis, we studied how large the influence of a conservative representation of the electrical flux in the computation of the secondary B-field is by adopting the DG-FEM is adopted. By comparing the DG-FEM with a conservative (Equation 39) and non-conservative (Equation 38) flux in a 4 mm multi-layer homogeneous sphere model, the high importance of DG-FEM with conservative flux could be worked out, outperforming the non-conservative DG-FEM scheme in all cases (Figure 5). In light of these results, the conservative flux DG-FEM was then used in all consecutive studies.

Results from the third study show the convergence of the DG-FEM numerical solutions toward the analytical solution when the resolution of the meshes is increased from 4 mm over 2 mm down to 1 mm (Figure 6).

From our comparison studies between CG- and DG-FEM regarding the secondary and the full B-fields, we first of all noticed that the accuracy for the 1 mm mesh resolution is extremely accurate for both methods: the mean RDM% is only up to ≈1.5% and the mean MAG% only up to ≈0.1% for sources with realistic eccentricities of 0.9796 (i.e., 1.59 mm to the next conductivity jump at the brain-CSF boundary) (Figures 7, 8). With ≈1.15% median RDM% and ≈0.01% MAG% for DG-FEM for the 1 mm mesh, also the IQRs are very low. The result is only slightly influenced by some few outliers, which showed higher numerical errors. However, these errors might be avoided by better controlling the source position with regard to the mesh, for example by only allowing sources in the center of the cortical hexahedra.

To the best of our knowledge, not many recent studies on finite element methods applied to solve the MEG forward problem have been presented, and none of them about DG-FEM. (Van den Broek et al., 1996) applied a CG-FEM approach in a 10 cm single-layer homogeneous sphere model and RDM% errors were measured. The minimum RDM% found by these authors for far less eccentric sources of 0.95 was 3%. Still, the comparison is not straightforward because of the different approaches, the different element meshes (tetrahedrons vs. hexahedrons), and the different source models which have been used. In general, tetrahedral meshes can better approximate surfaces but, for realistic head models, the generation of such models is difficult in practice and might cause unrealistic model features, e.g., holes in tissue compartments such as the foramen magnum and the optic canals in the skull are often artificially closed to allow constrained Delaunay tetrahedralization (CDT). Furthermore, CDT modeling necessitates the generation of nested, non-intersecting, and non-touching surfaces. However, in reality, surfaces might touch, for example, the inner skull and the outer brain surfaces. Hexahedral models, as investigated here, have larger geometry approximation errors, but do not suffer from the above limitations and can be easily generated from voxel-based MRI data. However, with new methods like in Nüßing et al. (2016), such geometry approximation errors can be avoided without the need of generating geometry conforming tetrahedral meshes.

van den Broek et al. (1998) used both a sphere model and a realistically shaped model. In both cases only three compartments were modeled, namely the brain, the skull and the scalp (brain and scalp with the same conductivity values, and skull with a 1:80 conductivity ratio). The authors used a lower amount of sources and lower mesh resolutions, but locally-refined tetrahedra meshes. In both scenarios, magnetometer sensors were covering only the top half of the models.

A CG-FEM MEG forward modeling study in a human (and rabbit) head volume conductor model was performed by Haueisen et al. (1995). The authors distinguished 12 or more homogeneous and isotropic realistically shaped head tissue compartments and used 2 mm FEM models. Since the focus was on sensitivity analysis and suppression ratio (i.e., the magnetic field of radial dipole divided by the one of the corresponding tangential dipole, was found to be in average 0.19 ± 0.07 in the realistic human head model) and not on validation in sphere models like in our study, we can not further compare these results to our results.

Another example of a CG-FEM and Biot-Savart's law scheme used to compute the electric potential and the B-field was presented by Schimpf et al. (2002). Similar to our approach, the authors used a 1 mm hexahedral mesh of a 4-layer piecewise homogeneous and isotropic sphere model. Also the arrangement of sources and sensors was similar to our work. The main focus of their work was, however, on source modeling: it was found that from the different tested source modeling approaches, the subtraction approach, also used in our study at hand, was the most accurate one.

In Vorwerk (2016, Ch. 2.10.4), for solving the MEG forward problem, three different CG-FEM source modeling approaches (i.e., subtraction, Saint Venant and partial integration) were compared in a 1 mm hexahedral (and in tetrahedral) meshes. Both the secondary and the full B-fields were examined against the analytical solution in a multi-layer homogeneous sphere model for tangentially oriented magnetometers. Also in this comparison it was found that the subtraction approach outperforms the other source modeling methods with regard to numerical accuracy for all sources apart from the most eccentric one. The subtraction method is therefore most sensitive to very close conductivity jumps and thus needs high resolution meshes especially in the source area, a result which is in line with ours. Deeper comparisons are again not easy because of different set-ups, but we are planning a direct comparison of the SimBio^3^ code used by Vorwerk (2016) and our DUNEuro implementation in future studies.

In Vorwerk et al. (2014), a guideline for EEG and MEG forward modeling using CG-FEM Saint Venant modeling was presented in realistic head models with a varying number of layers and conductivity profiles. The main result was that it is highly recommended to include the CSF and distinguish between gray and white matter and that, especially for the MEG, the modeling of skull spongiosa and compacta might be neglected. Furthermore, the numerical errors of a lower resolved (about 1 million nodes) 6 compartment anisotropic (6CA) model in reference to a higher resolved (about 2 millions nodes) version of 6CA were studied and expressed in terms of topography and magnitude errors: 95% of the sources had an RDM% of less than 2.5% and a MAG% of less than 10%.

Our last study was about the influence of leaky points on the computation of the secondary B-field when DG-FEM is adopted (Figure 9). In this analysis we considered two different multi-layer homogeneous sphere models, namely *seg*_*2*_*res*_*2* and *seg*_*2*_*res*_*2*_*r82*. The difference between the two models is that in *seg*_*2*_*res*_*2*_*r82* the thickness of the skull compartment is deliberately reduced so that 10,080 leaky points are present. When comparing CG- and DG-FEM in the leaky model (i.e., *seg*_*2*_*res*_*2*_*r82*) and in the non-leaky model (i.e., *seg*_*2*_*res*_*2*), we observed that the results of the computation of the secondary B-fields are almost identical. This means that the skull leakages neither cause additional MEG forward modeling errors for DG-FEM, nor for CG-FEM. The situation is thus different from the EEG case, where remarkable errors for CG-FEM forward modeling were shown, while DG-FEM could strongly alleviate these additional leakage errors (Engwer et al., 2017). For MEG, in case of tangential sources, the return currents mainly flow parallel to the inner skull surface in the close environment of the source, so that the leakages do not affect the overall MEG forward solution. We have to underline the fact that the results obtained in leaky scenarios are not to be confused with those where real holes of a certain diameter, e.g., from trepanation, are present in the skull compartment. The skull leakages investigated in Engwer et al. (2017) and, consequently, in this work are due to erroneous or, in general, poor representation of the skull compartment and not to real holes in the skull compartment. Lau et al. (2014) found that MEG signals are influenced by skull defects such as post-surgical skull openings. They examined the influence of skull holes in MEG signals via *in vivo* rabbit brains experiments, finding that the MEG signal amplitude reduced by as much as 20%, especially if the source is central under the skull defect. Their conclusion is that MEG source modeling requires realistic volume conductor head models that incorporate skull defects. Furthermore, Lau et al. (2016) showed that also MEG inverse solutions are affected by skull defects. In particular, ignoring skull defects in the head model during reconstruction displaced and reoriented sources under a skull defect, and when skull defects were incorporated in the head model with their physical conductivity, the location and orientation errors were mostly eliminated.

A further important aspect to discuss is that, if a combined EEG and MEG source reconstruction is strived for (Fuchs et al., 1998; Aydin et al., 2015), the same forward model should be used for both EEG and MEG, because of considerable advantages in terms of implementation, accuracy and computational cost efficiency, as the MEG forward model is also based on the electric potential and thus the numerical solution of the EEG forward problem. We therefore employed the same method (CG- or DG-FEM, with conservative or non-conservative flux representation) for both EEG and MEG in our work at hand. Accordingly, in case of EEG or combined MEG/EEG source reconstruction in possibly leaky head models (e.g., in temporal bone areas or, more generally, in children investigations), the usage of DG-FEM is recommended. In fact, DG-FEM clearly improves EEG forward solutions in leaky models (Engwer et al., 2017) and, at the same time, delivers reliable and accurate MEG solutions, as shown in the study at hand.

In this study, we did not evaluate the computational costs of the CG- and DG-FEM schemes for the computation of the MEG forward solution. Because of the higher number of degrees of freedom, DG-FEM is computationally more expensive than CG-FEM. However, the FEM transfer matrix approach (section 2.4) considerably reduces the computational costs of both approaches, so that this aspect gets less relevant for practical applications.

We now discuss possibilities for further accuracy increase that we plan to evaluate in our future work. In this study, sources were just chosen randomly, i.e., the influence of the source position relative to an element of the discretization was not yet investigated. It is well known that the combination of computing leadfields only for the most accurate sources combined with leadfield inter- and extrapolation techniques for other sources might not only speed up computations, but might also further increase numerical accuracy (Yvert et al., 2001; Vorwerk, 2011). In the DG-FEM scheme, indeed, already in the EEG forward computation (see Equation 26), the contribution given by the integral over Γ_*int*_ can reach high values when the source is relatively close to a quadrature point on the internal skeleton, because of the singularity in ∇*u*^∞^. Moreover, in Drechsler et al. (2009), an analytical expression for ∇*u*^∞^ was derived for isotropic and anisotropic conductivity distributions in the source space. A further future goal will thus be its implementation and use to further decrease the numerical errors in our FEM implementations, both on the CG- and DG-FEM sides. In addition, the degrees of polynomials in $V_{h}^{1}$ can be increased, together with the order of the Raviart-Thomas function space used to extend the conservative flux into the volume of each element. On the other hand, increasing the order of function spaces results in increased computational costs, so this intervention should be treated carefully. Furthermore, the DG-FEM constitutes the first step for the UDG-FEM implementation. This method, already tested in an EEG study (Nüßing et al., 2016), reduces the geometrical error of the forward simulations in hexahedral models while drastically decreasing the computational cost and thus its application to the MEG forward modeling represents an interesting future goal.

Overall the newly implemented conservative flux DG-FEM scheme offers an interesting new EEG and MEG forward modeling approach. It can be used especially in leakage scenarios and, in general, for comparison purposes, not only in EEG and MEG source analysis, but also in bioelectromagnetism applications, i.e., including also the simulation of transcranial electric and/or magnetic stimulation (Miranda et al., 2006; Datta et al., 2013; Windhoff et al., 2013; Wagner et al., 2014).

## 6. Conclusions

We presented theory, validation and evaluation of three finite element method (FEM) approaches for the MEG forward problem, namely the continuous Galerkin FEM (CG-FEM), as well as two new approaches, the discontinuous Galerkin FEM (DG-FEM) with a conservative and a non-conservative flux implementation. All three methods have been implemented in the DUNEuro software module. Statistical validations and evaluations have been performed on multi-layer homogeneous sphere models represented via hexahedral meshes and the subtraction approach has been adopted as source model. DG-FEM with conservative flux implementation, i.e., a main feature of a DG-FEM discretization, turned out to be superior to the non-conservative flux variant. The new DG-FEM method showed proper convergence behavior with increasing mesh resolution. When compared to the CG-FEM, DG-FEM provided results that are in a comparable range of high accuracy. Furthermore, both methods are able to model realistic head volume conductor models with their tissue inhomogeneities and anisotropies. In contrast to EEG studies, the so-called skull leakage effects did not play a crucial role for MEG. However, for EEG or combined MEG/EEG source analysis scenarios, DG-FEM offers an interesting new alternative to CG-FEM, considering the importance of a high accuracy of the forward problem solution in MEG/EEG source reconstruction. Finally, the DG-FEM MEG forward simulation in a realistic head model for an auditory source resulted in EEG and MEG topographies that are in line with practical findings in the field of auditory evoked responses.

## Author contributions

MP, CE, and CW conceived the study. MP wrote the code with the supervision of AN and CE. JV and CW provided spherical and realistic head models. MP and JV constructed the FEM models. MP performed the simulations and the analysis, produced the images, interpreted the results, and wrote the paper. All authors took part in the scientific discussion at multiple stages of the study and provided feedback from the modeling (CW, CE, HB, RO), theoretical (CE, CW, AN, JV), and technical (AN, CE) perspective. All authors reviewed the manuscript and approved it for publication.

### Conflict of interest statement

The authors declare that the research was conducted in the absence of any commercial or financial relationships that could be construed as a potential conflict of interest.

## Footnotes

^1^http://www.dune-project.org

^2^www.duneuro.org

^3^https://www.mrt.uni-jena.de/simbio/index.php/Main_Page
